# MRI of a recurrent adenoid cystic carcinoma of the trachea, treated with fast neutron therapy

**DOI:** 10.1259/bjrcr.20150201

**Published:** 2016-05-18

**Authors:** Laura Sweeney, Frederik Vernimmen, Sandra Sinske

**Affiliations:** ^1^ Department of Radiation Oncology, Cork University Hospital, Cork, Ireland; ^2^ University of Stellenbosch/iThemba LABS, Cape Town, South Africa

## Abstract

Adenoid cystic carcinoma (ACC) of the trachea is a rare tumour, which responds well to high linear energy transfer radiation, such as neutron therapy. Both CT and MRI are useful for its diagnosis, with MRI being superior at determining the extent of disease and perineural involvement. Identification of these disease characteristics with MRI helps to determine lesion resectability and decide on the most appropriate treatment strategies. MRI is also useful in the differentiation of post-radiation change from disease recurrence, with post-radiation change displaying low *T*_2_ signal intensity compared with tumour recurrence displaying intermediate to high *T*_2_ signal intensity. Furthermore, MR diffusion-weighted imaging can be useful in the distinction between the two. We present a case of tracheal ACC treated with fast neutron therapy and followed with MRI.

## Summary

Primary tracheal carcinoma is rare, with an incidence of 0.2 per 100,000 persons per year and a prevalence of 1 per 15,000 autopsies.^1^ Of all primary tracheal carcinomas, the most common histology is that of squamous cell carcinoma, followed by adenoid cystic carcinoma (ACC), thereby making ACC a rare tracheal tumour.^2^

ACC most commonly arises in the major and minor salivary glands, but in the trachea it arises from the mixed seromucinous glands in the submucosa of the tracheobronchial tree.^3^

ACC has been described as a slow growing tumour with a long natural history and its insidious nature is often the reason for delay in seeking early medical consultation. It can be persistent and recurrent, with late onset of metastases.^4^ ACC is three times more likely to extend beyond the trachea than squamous cell carcinoma, so larger margins of clearance are required for both surgical excision and radiation therapy.^5^

The treatment of choice for tracheal carcinoma is primary resection and post-operative irradiation. For unresectable disease, or for patients in whom surgery would cause considerable morbidity, initial primary treatment with fast neutron therapy (FNT) has been found to offer high local–regional control and survival rates.^6^

Finding a radiological standard for evaluation of tracheal ACC can be challenging, as different modalities have certain different advantages. For diagnosis, CT is helpful at demonstrating the luminal extent of the tumour and its relationship to the surrounding structures.^7^ On the other hand, MRI can accurately identify the tumour depth and invasion in different planes, providing information that is helpful for treatment planning.^8^ For follow-up post radiotherapy, MRI is more helpful in differentiating recurrence from post-radiotherapy fibrosis.^9^ We describe a case where we used MRI for recurrence evaluation, treatment planning and follow-up of recurrent ACC of the trachea treated with FNT.

## Case presentation

DC, a 51-year-old male, underwent complete resection of a tracheal lesion in 1981. The lesion was 9 cm below the vocal cords and 4.5 cm superior to the carina. It measured 4.5 cm in length, and a total specimen length of 7 cm was resected (allowing for margins) *via* midline sternotomy, followed by end-to-end anastomosis. Histology demonstrated an ACC. He did not undergo adjuvant therapy.

**Figure 1.**
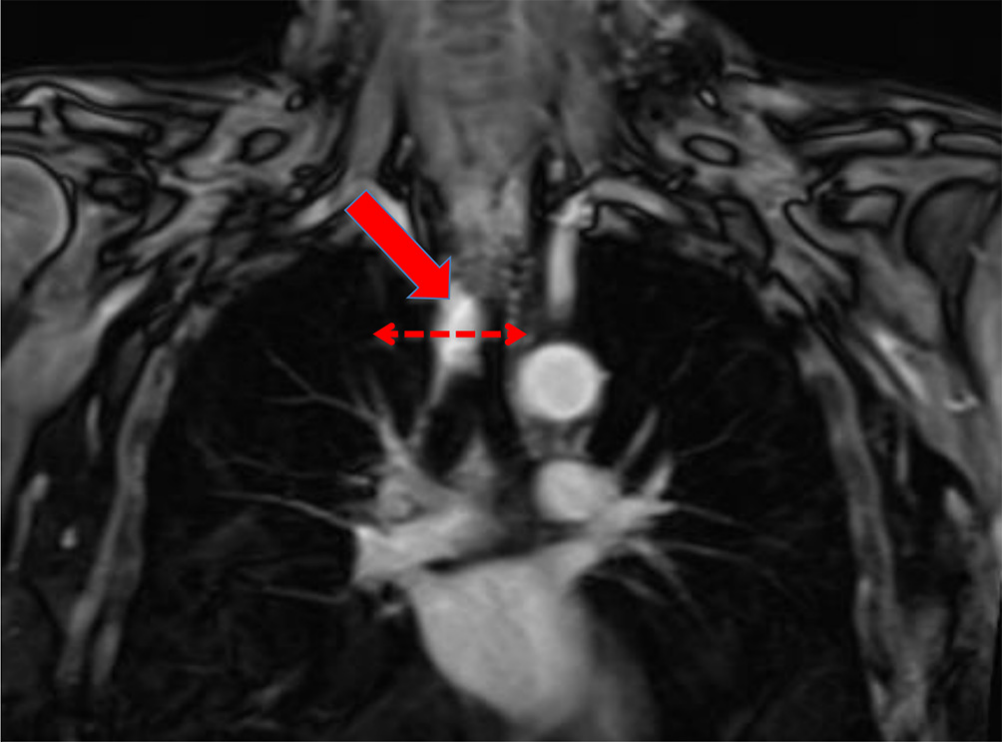
Contrast-enhanced MRI of the mediastinum: coronal slice. Block arrow: recurrent tumour, pre-treatment (November 2011). Broken arrow: level of previous surgical resection and end-to-end anastomosis.

**Figure 2.**
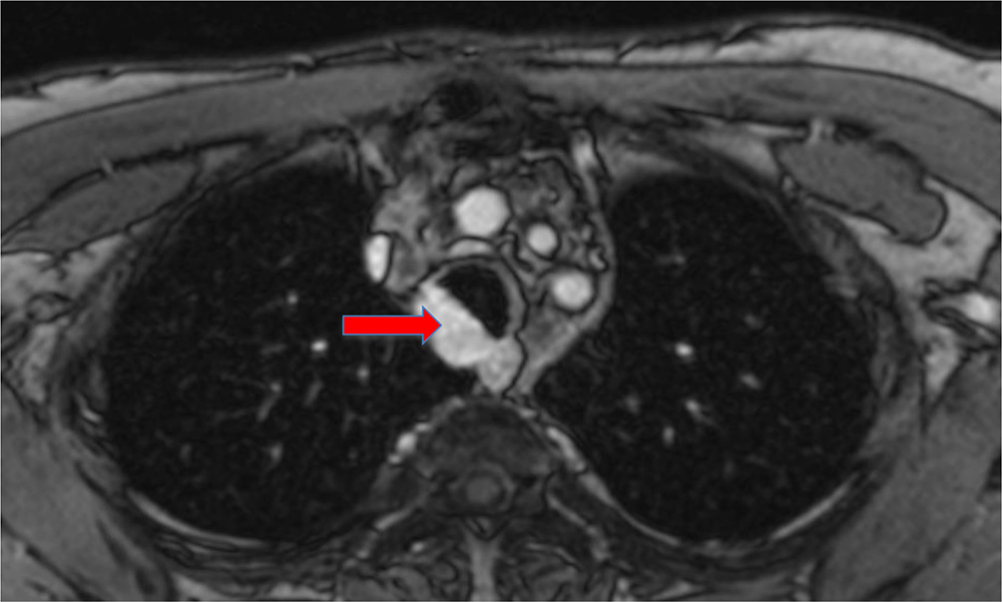
Contrast-enhanced MRI of the mediastinum: axial slice. Block arrow: recurrent tumour, pre-treatment (November 2011).

In March 2011, DC presented with symptoms of a lower respiratory tract infection. Investigations, including CT/MRI of the thorax, demonstrated a mass in the trachea (Figures 1 and 2). Bronchoscopy demonstrated a smooth, lobulated lesion on the right posterolateral wall of the trachea with a 25% cross-sectional encroachment of the trachea. Biopsy and histology confirmed local recurrence of ACC. A positron emission tomography/CT (PET/CT) scan showed low-grade avidity in the tumour area with no evidence of metastatic disease.

**Figure 3.**
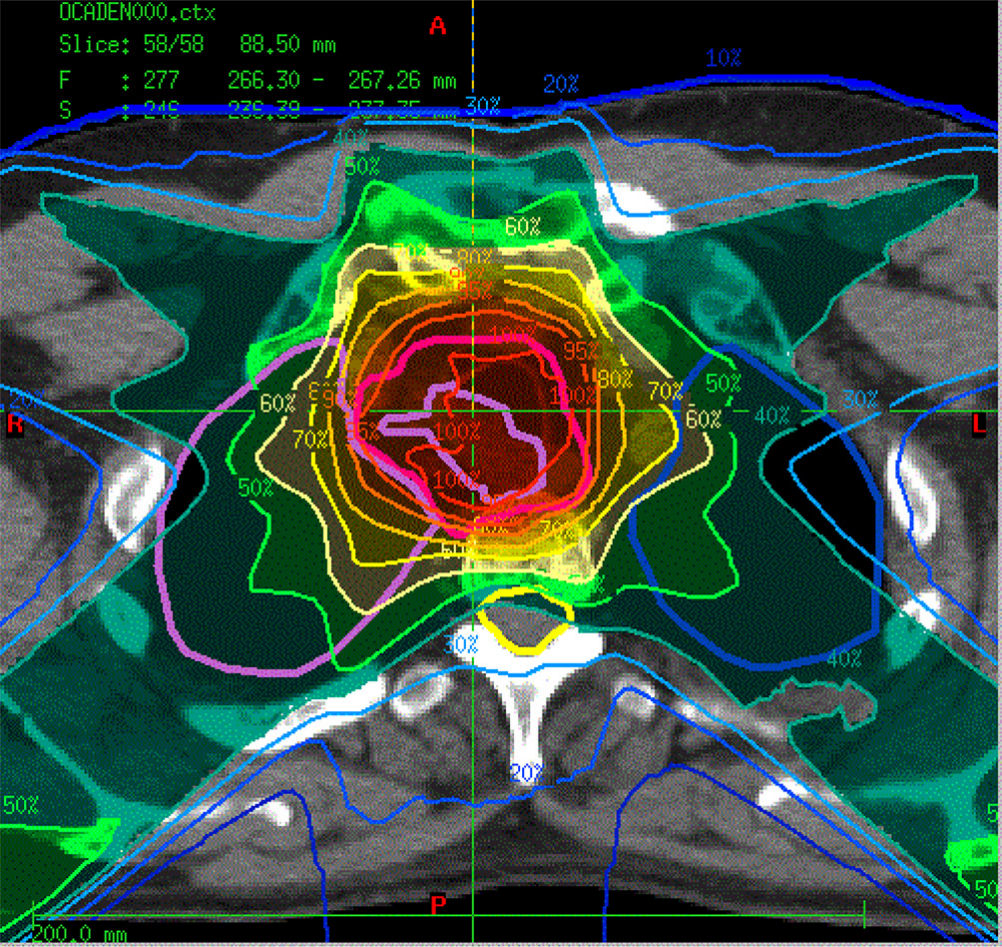
Isodose demonstrating coverage of at least 95%, 5 mm from the clinical target volume.

After extensive multidisciplinary discussion and wide surgical consultation, the lesion was considered unresectable and therefore the patient was considered for primary radiation therapy. In light of the histology, it was felt that the best radiotherapy approach would be with FNT and so DC received a standard curative dose of 20.4 Gy in 15 daily fractions given three fractions per week from November until December 2011. The relative biological effect (RBE) of neutrons is dependent on the way they are produced.^10^ At iThemba LABS (Cape Town, South Africa), where the patient was treated, an RBE of 3 for normal tissue has been used for all treatments given on the p(66)/Be isocentric unit.^11^ Hence the equivalent photon dose to the normal tissue was 61.2 Gy. For the dose plan, the gross tumour volume (GTV) was delineated and the clinical target volume (CTV) included circumference of the trachea combined with a 5-mm margin around the GTV. The planning target volume (PTV) was obtained by expanding the CTV by 5 mm in all directions (Figure 3).

**Figure 4.**
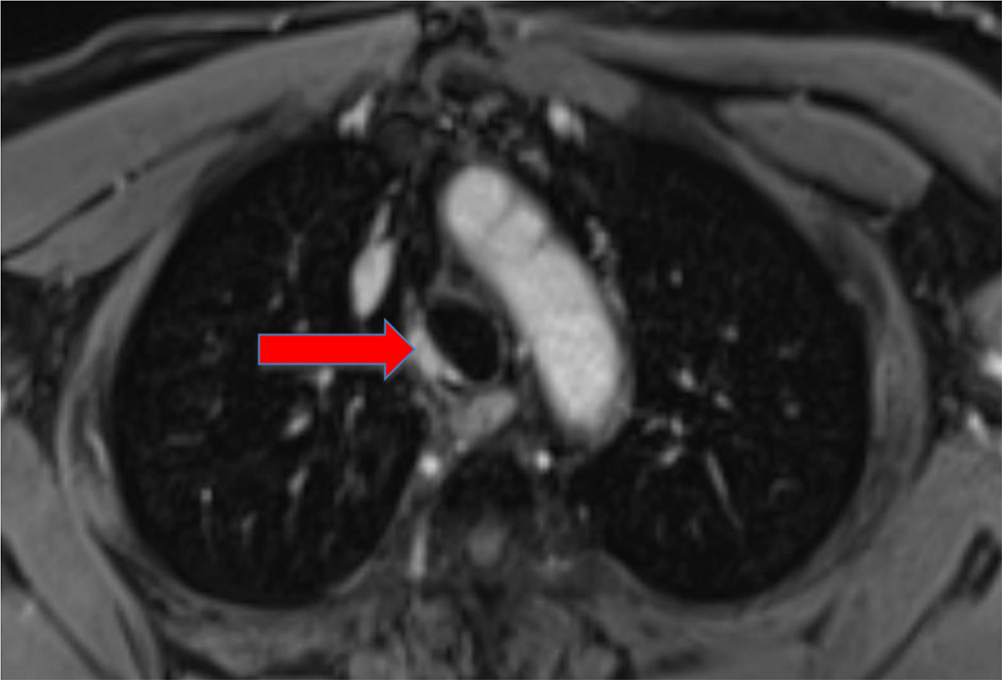
Contrast-enhanced MRI of the mediastinum: axial slice. Block arrow: reduction in tumour size, 1 year post-treatment (November 2012).

**Figure 5.**
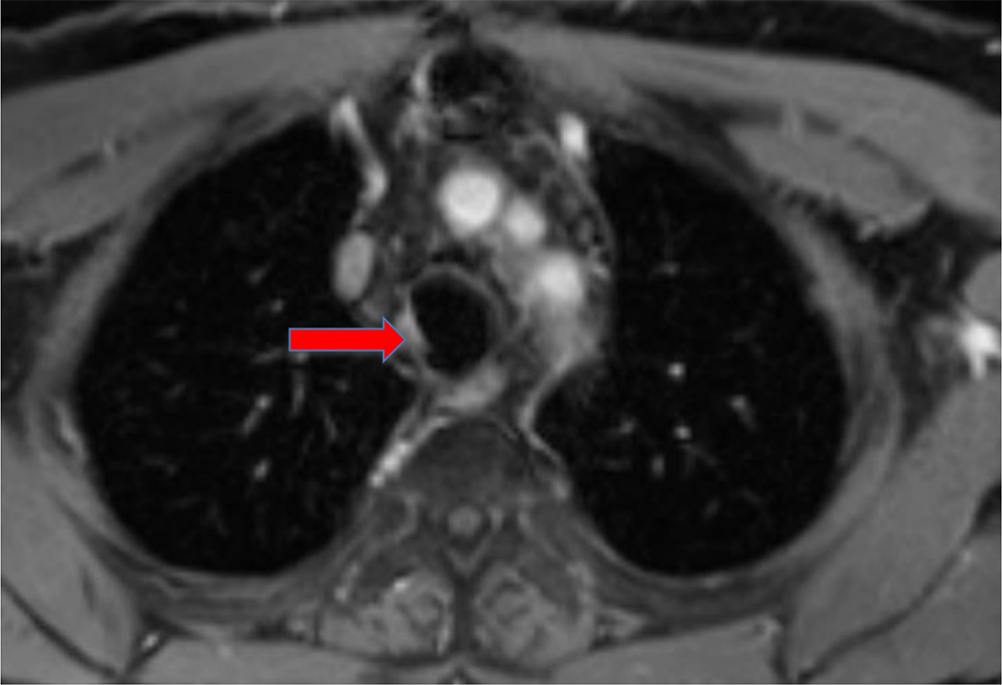
Contrast-enhanced MRI of the mediastinum: axial slice. Block arrow: further reduction in tumour size, 3 years post-treatment (November 2014).

Follow-up consisted of regular clinical examinations and 6-monthly MRIs for the first year, and subsequent yearly MRIs. He also underwent an oesophagogastroduodenoscopy in 2014, which was normal. These serial MRIs showed a gradual disease response with reduction in the size of the ACC (Figures 4 and 5). This response to neutron therapy is characteristic for this pathology. Clinically, the patient is doing very well and is not reporting any late side effects of the treatment.

## Discussion

FNT has been shown to be beneficial over photons in the treatment of salivary gland tumours.^6^

Neutrons are a form of high linear energy transfer (LET) radiation, depositing about 20–100 times more energy than photon radiation.^12^ High LET radiation causes more direct damage to the DNA in comparison with the “indirect” damage photons *via* free radicals. Therefore, there is less dependency on oxygenation status, with the oxygen enhancement ratio for neutrons being approximately half that of photons.

Neutron damage is usually single-hit, double-stranded DNA breaks that are very difficult for tumour cells to repair. In contrast lower LET radiation is not as damaging, because the single-strand DNA breaks are easier for tumour cells to repair. High LET radiation is also less cell cycle dependent for cell damage.^13^

These LET characteristics incur a different RBE compared with photons. Neutrons have been demonstrated to have a high RBE in the treatment of ACC. Battermann et al^14^ reported an RBE of 8 for metastatic ACC, compared with an RBE of 3–3.5 for normal tissues. This is a gain of 2.5 over conventional photon therapy, accounting for the improved outcome of neutron therapy in ACC.^14^

CT has traditionally been considered the modality of choice for airway disease. CT is helpful in demonstrating the intraluminal and extraluminal extent of the disease, has excellent spatial resolution, shorter imaging time, is less expensive and more readily accessible, when compared with MRI.^9^ However, disease evaluation with CT is limited owing to its inability to define the extent of submucosal infiltration. MRI has the ability to assess this, thereby allowing more effective planning prior to surgical intervention or radiotherapy.^15^

As shown in Figures 1,2,4 and 5, MRI has been very useful in our case for pre-treatment assessment and evaluating therapeutic response in terms of possible disease recurrence and radiation-induced changes.

MRI characteristics of ACC include an irregular contour, poorly defined margins, low to intermediate *T*_1_ signal, homogenous enhancement on contrast enhanced *T*_1_, and high signal intensity on *T*_2_ weighted images. MRI in multiple planes and sequences shows the different signal intensities of tumour, fat, fluid and nerve, allowing the evaluation of the extent of tumour invasion into adjacent structures.^8,16^

ACC has a high propensity for perineural spread, and this characteristic is an important factor in treatment planning and prognosis. Detection of perineural involvement prior to initiation of treatment is important, as many treatment failures are due to unidentified perineural spread.^17^ While both CT and MRI can detect perineural involvement, MRI is better owing to its superior soft tissue delineation, whereby replacement of perineural fat with tumour, enhancement with gadolinium and increased nerve size are characteristics of perineural spread.^18^

During follow-up after high LET therapy, tumours can seem slow to regress and it may take some time before a reduction in size is noticed radiologically. Hence, care should be taken not to make the assumption of poor treatment response or disease recurrence too early in the follow-up period. MRI can help differentiate tumour recurrence from post-treatment changes within this treated area. Tumour recurrence generally enhances on contrast enhanced *T*_1_sequences and demonstrates intermediate to high *T*_2_ signal. Post-radiotherapy change also enhances on contrast enhanced *T*_1;_ however, it typically displays low *T*_2_ signal indicative of fibrosis and scarring. Early post-radiation inflammatory change can result in a high *T*_2_ signal, but again care should be taken not to assume this is disease recurrence. As in our case, the first follow-up MRI is generally 6 months post therapy, which allows time for early post-therapy oedema to settle. Recurrence and post-treatment change can be further distinguished using MR diffusion-weighted imaging. Using diffusion-weighted imaging, recurrence can be seen to display a low apparent diffusion co-efficient (ADC), reflecting the restriction of movement of water molecules in the extracellular space due to tumour hypercellularity. On the other hand, due to the relative hypocellularity of scar tissue, post-radiation change tends to display a high ADC value.^19^

## Learning points

1. Both CT and MR are useful in the diagnosis of tracheal ACC, with MRI being superior in the assessment of local invasion. This in turn helps determine the resectability of the lesion.
2. MRI assessment of perineural invasion enables more accurate delineation of radiotherapy target volumes.
3. MRI is superior to CT for follow-up, as it allows differentiation between tumour recurrence and post-radiotherapy change.
4. Slow radiological tumour regression after high LET particle therapy is typical for ACCs, and care should be taken not to make the diagnosis of a recurrence too early in the follow-up period.

## Consent

Written informed consent for the case to be published (including images, case history and data) was obtained from the patient for publication of this case report,
